# Multidimensional materials and device architectures for future hybrid energy storage

**DOI:** 10.1038/ncomms12647

**Published:** 2016-09-07

**Authors:** Maria R. Lukatskaya, Bruce Dunn, Yury Gogotsi

**Affiliations:** 1 Department of Materials Science and Engineering, A.J. Drexel Nanomaterials Institute, Drexel University, Philadelphia, Pennsylvania 19104, USA; 2 Department of Materials Science and Engineering, University of California, Los Angeles, California 90095, USA

## Abstract

Electrical energy storage plays a vital role in daily life due to our dependence on numerous portable electronic devices. Moreover, with the continued miniaturization of electronics, integration of wireless devices into our homes and clothes and the widely anticipated ‘Internet of Things', there are intensive efforts to develop miniature yet powerful electrical energy storage devices. This review addresses the cutting edge of electrical energy storage technology, outlining approaches to overcome current limitations and providing future research directions towards the next generation of electrical energy storage devices whose characteristics represent a true hybridization of batteries and electrochemical capacitors.

## Abstract (web summary)

With the continued miniaturization of electronics, there are increasing efforts to engineer small, powerful energy storage devices. Here the authors review the cutting edge of this rapidly developing field, highlighting the most promising materials and architectures for our future energy storage requirements.

## Graphical abstract

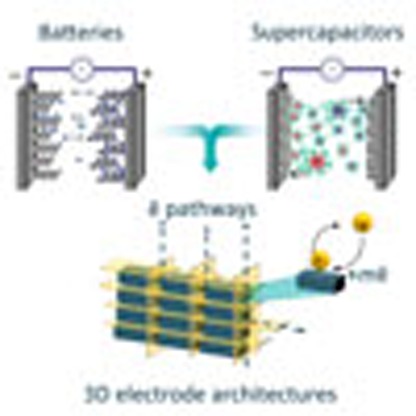

Achieving a secure, sustainable energy future is one of the greatest scientific and societal challenges of our time. Electrical energy storage (EES) plays a vital role in daily life because of our dependence on numerous electronic devices that require mobility. There is also a need for large-scale and inexpensive EES for grid and transportation applications.

Electricity generated from renewable sources, such as solar and wind, is critical to meeting future energy demands. However, these intermittent renewable sources require efficient EES devices. Rechargeable (secondary) batteries, which use electrochemical reactions for energy storage, are commonly used for EES at small and medium scales^1^. Lithium-ion batteries currently dominate the market for electronic devices, and are rapidly penetrating the transportation segment and entering into grid scale storage. A multitude of other batteries are available commercially, including well known lead-acid batteries. However, modern technologies require much larger amounts of energy to be stored with a high energy density, quickly and at low cost. Electrochemical capacitors (ECs) can be charged in minutes if not seconds, ensuring fast energy collection, but they store 1–2 orders of magnitude less energy compared with batteries^2^. Thus, the development of new EES systems is needed in order for large-scale solar- and wind-based electrical generation to be practical^3^,^4^. The time it takes to charge current batteries is long compared with what it takes to fuel our gasoline vehicles. Therefore, substantial improvements in the energy and power densities of EES systems are also needed to realize the electrification of transportation. Moreover, with the continued miniaturization of electronics, penetration of wireless devices into our homes and clothes, wide use of sensor networks and widely anticipated ‘Internet of Things', there is an intensive effort to develop miniature, but powerful EES devices. Current thin-film batteries have a very small amount of active material relative to the packaging and other passive components. EES that have a much smaller content of passive materials have a very long cycle life and can be manufactured on any surface as required^1^,^2^,^3^,^4^.

The limitations of current EES devices expose fundamental gaps in our understanding of atomic- and molecular-level processes that govern their operation, performance and failure. This article reviews the development of a new generation of sustainable, affordable and safe EES technologies that approach the theoretical limit for electrochemical storage and deliver electrical energy rapidly and efficiently. Here, the ‘theoretical limit' refers to the limit imposed by thermodynamic considerations, rather than limitations coming from electrochemical system design. First, mechanisms of electrochemical energy storage are discussed, followed by a description of energy storage in asymmetric and hybrid devices, where each electrode in the cell utilizes a different mechanism of charge storage. Hybrids of batteries and ECs represent an important future direction for EES, where there is the promise of achieving high energy and power densities, well beyond what is anticipated from improvements in lithium-ion batteries or carbon-based electrical double-layer capacitors (EDLC). To achieve this goal, there is a need for novel materials and system architectures, which enable electron transfer and ionic transport with multi-electron redox chemistries, as stressed in a recent review^5^. In appraising the status of this research, it is important to highlight that future opportunities lie in the computationally driven design of new materials and hybrid energy storage devices. Finally, some gaps in the understanding of atomic- and molecular-level processes that govern operation, performance and failure of EES devices are highlighted and pathways to the integration of faradaic and capacitive storage mechanisms are outlined.

## Faradaic and capacitive energy storage

**Figure 1:**
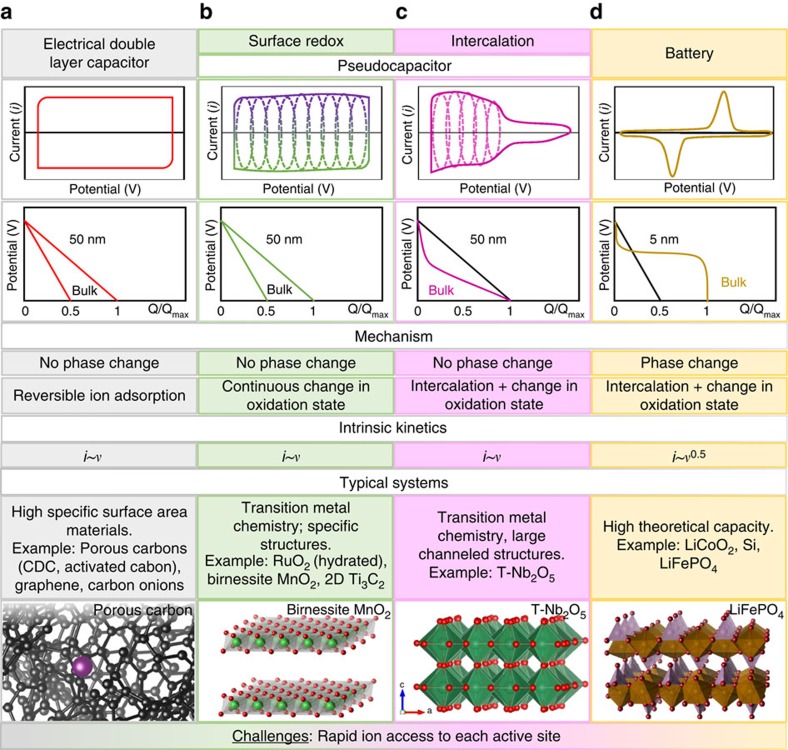
Faradaic and capacitive energy storage. Summary of the characteristic metrics such as cyclic voltammetry, galvanostatic profiles, key mechanism descriptions and typical systems that are known to utilize the mentioned charge storage mechanisms: (**a**) double-layer capacitor (examples: porous carbons (carbide derived carbon^8^, activated carbon^91^), graphene^44^, carbon onions^40^ and nanotubes^92^), (**b**) surface redox pseudocapacitance due to adsorption and/or fast intercalation of ions (examples: hydrated RuO_2_ (ref. 93), birnessite MnO_2_ (ref. 12), MXene Ti_3_C_2_ (ref. 15)), (**c**) intercalation pseudocapacitance (example: T-Nb_2_O_5_ (ref. 17)) and (**d**) batteries (examples: LiCoO_2_ (ref. 94), Si^37^, LiFePO_4_ (ref. 1)). *i*=current, *v*=sweep rate. Crystal structures were plotted in Vesta. Different colours in the plots indicate different storage mechanisms.

Understanding the operative mechanisms of energy storage systems is extremely important, not only from a fundamental point of view, but also because knowledge of ion storage and transport mechanisms builds a strong basis for the development of devices and their resulting performance. Figure 1 illustrates the characteristic behaviour of these electrochemical energy storage materials and summarizes the features that distinguish them from each other.

Supercapacitors or ECs represent a class of energy storage devices that offer fast energy uptake and delivery^2^. EDLCs utilize reversible ion adsorption at the surface or inside pores to store charge. Thus, the best performing EDLC materials should have a high specific surface area (SSA), making nanostructured carbons the materials of choice, since they can easily deliver high SSA at low price and possess good electrical conductivity. Generally, EDLCs offer great cyclability and power densities and are characterized by nearly rectangular cyclic voltammograms (CVs) and linear galvanostatic charge–discharge profiles (Fig. 1a). It is important to note that the rectangular CV for EDLCs stems from the fundamental nature of the double-layer storage mechanism: in the absence of diffusion limitations, there is instantaneous charge separation (that is, polarization) in a capacitor under an external electrical field so that d*V*/dt is a constant. While ideal for power applications, EDLCs suffer from limited energy density (the amount of charge stored).

For the most part, it is clear what EDLCs are and how they differ from batteries and pseudocapacitors, both of which use faradaic reactions to store charge^6^. There are important considerations for microporous activated^7^ and carbide-derived carbons (CDC)^8^ with average pore diameters of about 1 nm. These materials can usually accommodate one ion or a few ions per pore, since ions can lose their solvation shell in part or entirely while entering the pores^8^. Although charge storage in these materials is purely electrostatic, they are traditionally called EDLCs, despite the fact that no classical Gouy–Chapman–Stern double layer is formed. A more accurate representation is to consider their charge storage process as arising from the electrosorption of ions by an oppositely charged carbon sponge, similar to physisorption of molecules by the same porous carbons, but driven by electrostatic force. It was shown that a theoretical model that more accurately describes this effect is the electric wire-in-cylinder capacitor instead of a parallel plate capacitor^9^.

More confusion arises when researchers try to distinguish between batteries and pseudocapacitors, even though guidelines for distinguishing between these two types of energy storage materials have been proposed^6^,^10^. The aim of this review is not only to highlight the distinctions, but also to elaborate about what makes them different. In this regard, two key features are phase transformations and kinetics, as outlined below.

### Phase transformations

Charging in batteries is often accompanied by a phase transformation of the host material. This process is characterized by distinct peaks in the CV and plateaus in the galvanostatic charge–discharge profiles (Fig. 1d). In contrast, charging of the pseudocapacitor should not be accompanied by a phase transition. Instead, pseudocapacitive materials present a continuous, highly reversible change in the oxidation state during charge/discharge, characterized by CVs with either significantly broadened peaks (intercalation, Fig. 1c) and little separation in peak position on charge/discharge, or almost perfectly rectangular CVs (surface redox, Fig. 1b)^2^,^11^.

### Intrinsic kinetics

While there are many factors that can contribute to the efficiency of charging (Q/Q_max_) at different rates, the sweep rate dependence for CV experiments provides insight regarding the kinetics for intrinsic electrochemical processes. The current (mA), *i*, in battery electrode materials exhibits classic semi-infinite diffusion (that is, *i∼v*^*0.5*^), while supercapacitors are characterized by a linear sweep rate dependence on current, *i∼v*.

The connection between these two features is that there are both structural and kinetic considerations associated with phase transformations. Dimensional changes arising from ion insertion will often lead to strains; phase transformations occur to relax the strain. In addition, phase transformations may result in significant volume changes, which negatively impact the integrity of the electrodes, and therefore cycling of the device is poor. Phase transformations generally involve nucleation and growth processes which influence kinetics, as non-martensitic (displacive) phase transformations are diffusion-limited. This is one reason why the rate response for battery materials is typically poor. However, when the crystallographic pathways for ion transport are interconnected and sufficiently large to accommodate mobile ions, some of the limitations for battery materials can be overcome.

**Figure 2:**
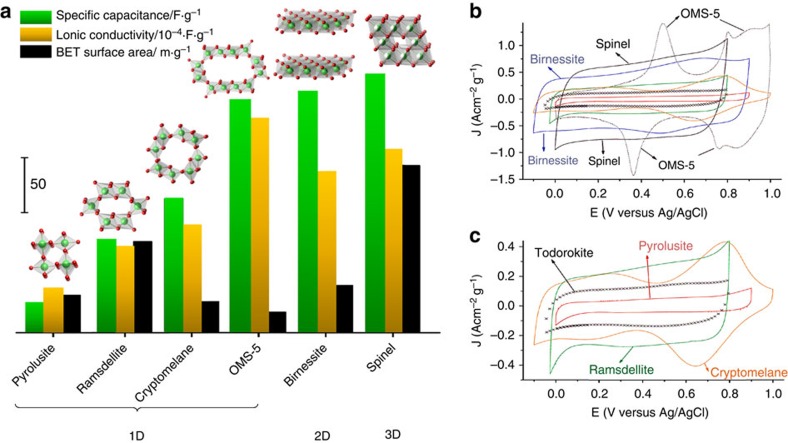
Capacitance of MnO_2_ allotropic forms. (**a**) Relative values of the specific capacitance, ionic conductivity and SSA of different MnO_2_ forms with 1D, 2D and 3D pore channels. (**b**,**c**) CV profiles collected in 0.5 M K_2_SO_4_ at 5 mVs^−1^ show distinctly different behaviour as a function of structure for materials of the same chemical composition (MnO_2_), but having different crystal structures with different sizes and shapes of crystallographic channels. Adapted from ref. 12 (Copyright 2009 American Chemical Society).

The importance of crystallographic pathways was nicely illustrated in a detailed study of the electrochemical behaviour of MnO_2_ allotropic phases with different characteristic geometries (Fig. 2a): one-dimensional (1D) channels, two-dimensional (2D) layers and three-dimensional (3D) interconnecting channels^12^. Since the chemical composition was fixed, the effect of structural and chemical parameters was evaluated. It was shown that an increase in channel size and connectivity resulted in improved electrochemical performance (Fig. 2). SSA had a marginal effect on the resulting capacitance, presumably because charge storage occurred from redox processes and not from the EDL. Interestingly, strong correlation was found between capacitance and ionic conductivity of different MnO_2_ phases. Ionic conductivity is directly influenced by the crystal structure of the material and, when aqueous electrolytes are utilized, the presence of structural water^13^,^14^. Conclusions from this study can be extended beyond MnO_2_, highlighting the crucial importance of material architecture. Due to the reasons noted above, it is possible to identify structural features, such as a narrow distribution of straight pores, a good match between the pore size and ion size, interconnectivity of pores and accessibility of redox-active sites, which are required for maximizing performance. Materials possessing these features offer considerable promise for energy storage applications: (i) 2D materials that contain transition metals (such as layered transition metal oxides^12^, carbides^15^ and dichalcogenides^16^) and (ii) materials with 3D interconnected channels (such as T-Nb_2_O_5_ (ref. 17 or MnO_2_ spinel^12^). Combinations of different mechanisms described above in EES devices lead to improved rate performance and the ability to store and deliver larger amounts of energy over extended periods of time, as described in the following section.

## Asymmetric and hybrid devices

### Asymmetric devices

**Figure 3:**
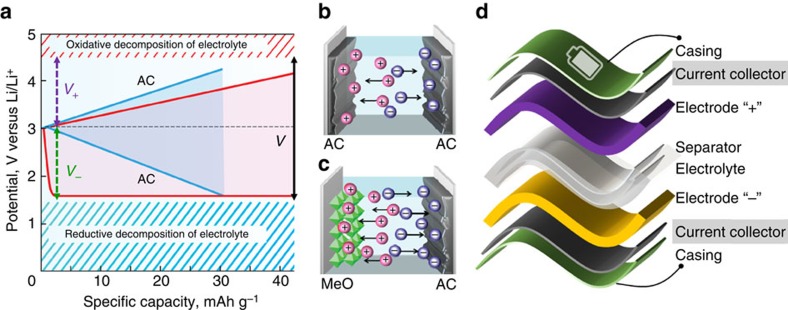
Hybrid versus symmetric electrochemical capacitors. (**a**) Schematic plot of the electrode potentials (*V*_*+*_ and *V*_−_) and cell potential (*V*) versus specific capacity for symmetric (blue lines) and hybrid (red lines) configurations. (**b**) Typical symmetric configuration featuring activated carbon (AC) as both positive and negative electrodes. (**c**) Example of hybrid device consisting of an insertion metal oxide (MeO) negative electrode (anode) combined with a high surface area carbon positive electrode (cathode) such as AC. Panels (**a**–**c**) were reproduced from ref. 21 (Copyright 2013 American Chemical Society). (**d**) Components of typical energy storage cell.

The definition of an asymmetric energy storage cell is very broad as it refers to every combination of positive and negative electrodes whenever there is any difference between the two electrodes (weight, thickness, material and so on). Thus, in designing supercapacitors, it is essential to recognize that a symmetric device (when both electrodes are identical, that is made from the same material and have same mass, thickness and so on) might not be optimal. For example, it is possible to expand the operating potential window, *V*, by using negative and positive electrodes made of different materials, as in a battery (Fig. 3a). The energy density of a device can be described by the well-known equation: 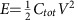, where *C*_*tot*_ is the capacitance of the device and *V* is the operating potential window of the cell. Other important considerations for charge storage are that (i) *1/C*_*tot*_*=1/C*_*+*_*+ 1/C*_−_, where *C*_+_ and *C*_−_ are corresponding capacitances (in Farads) of the positive and negative electrodes, (ii) *Q*_*+*_*=Q*_−_ for the cells with ∼100% coulombic efficiency, where *Q*_*+*_*=C*_*+*_*V*_*+*_ and *Q*_−_*=C*_−_*V*_−_ are charges stored on the positive and negative electrodes, respectively and (iii) *V*_*+*_ and *V*_−_ are the respective potential windows at positive and negative electrodes that are limited by irreversible processes, such as decomposition of electrolyte and over-reduction or over-oxidation of the electrode material, also *V=V*_*+*_*−V*_−_, as shown in Fig. 3a^18^. These fundamental equations provide the basis for characterizing asymmetric cell configurations.

Even for typical EDLC materials there is an asymmetry in the electrochemical behaviour for the cations and anions due to the difference in ion sizes with respect to pore diameter^19^. This results in unequal specific capacitances (in Farads per gram) for positive (*c*_*sp+*_) and negative electrodes (*c*_*sp*−_); that is, *c*_*sp*−_*≠c*_*sp+*_. Therefore, to maintain the stable voltage condition of *V*_−_*=V*_*+*,_ the weights of the electrodes (*m*_−_ and *m*_*+*_) should be unequal to compensate for the different specific capacitance values: 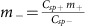. Since the current response for pseudocapacitive or battery materials varies with applied potential (Fig. 1c,d), the general case of combining two different electrochemical materials will produce an asymmetric energy storage cell.

It is important to note that for many applications, in particular for portable electronics and vehicles, volumetric energy density is more important than gravimetric energy density. In this case, to balance the performance of electrodes in asymmetric cells, similar considerations are applied, but instead volumetric capacitance (Farads per cubic centimetre) and electrode volume are used rather than gravimetric capacitance (Farads per gram) and mass. Volumetric and gravimetric performance are connected through electrode density, and to a large extent are defined by electrode design and porosity^20^.

### Hybrid devices

Hybrid devices constitute a special case of asymmetric cells. In hybrid devices, different charge storage mechanisms are implemented in the positive and negative electrode materials (Fig. 3a–c). For example, one electrode utilizes the double-layer storage mechanism (that is, porous carbon) while the other stores charge by means of faradaic reactions (that is, as occurs in transition metal oxides)^2^. The concept of asymmetric cells is very compelling, allowing us to produce an ‘ideal' combination of positive and negative electrode materials and to perform ‘smart' balancing of their weight/volume, so that overall device performance can be improved significantly in terms of voltage window, capacitance (and therefore energy density) and longevity in comparison with their symmetric counterparts^21^.

### Whole cell design

Apart from positive and negative electrodes, each energy storage cell/device contains electrolyte and a separator (to prevent short circuit between electrodes), current collectors and casing (Fig. 3d). Therefore, for many state-of-the-art energy storage devices, especially small ones, the weight of the overall device is 5–10 times the total weight of the positive and negative electrodes due to combined contributions of all device components^20^. Thus, to minimize the weight/volume of the inactive materials, several approaches can be applied:

(i) The use of thicker electrodes which maximizes material loading with respect to the entire device weight/volume. Therefore, development of material architectures that can provide high energy densities for the thicker electrodes is very important. This is one of the principal motivations for developing 3D composite electrode architectures that would address issues of impeded ion transport and decreased conductivity for thicker electrodes. Some initial examples demonstrating thicker structures include oxide nanoparticles supported on 3D graphene networks^22^, carbon nanotubes^23^ and graphene layers^24^,^25^ aligned normally to the separator surface.

(ii) The development of free-standing, highly conductive electrodes to eliminate current collectors. Achieving the electrode-as-current collector approach is possible when nanomaterials with metallic conductivity such as carbon nanotubes^26^, graphene^27^ or 2D metal carbides^15^ are used as active materials, or at least constitute a large part of the electrode and are well interconnected. It is worth noting that complete elimination of the current collector for large-scale high-power devices can be challenging due to insufficient conductivity of the commonly used conductive carbons (multi-wall carbon nanotubes or reduced graphene oxide). Therefore, either some other materials with higher conductivity should be used (for example, metallic single wall carbon nanotubes, transition metal nitrides/carbides and so on) or a compromise should be reached between the current collector thickness and power characteristics of the device.

(iii) The use of solid and gel electrolytes^28^, which eliminate the need for a separator, minimize the volume and improve mechanical properties of the overall device. This approach becomes especially significant in manufacturing flexible or microscale devices.

## Fully integrated rechargeable energy storage devices

In this section the most successful examples of hybrid devices are reviewed, especially hybrid ECs since they can provide excellent performance at high charge/discharge rates and represent important directions for further development.

As described above, a typical hybrid EC consists of two electrodes that use different charge storage mechanisms: a porous-carbon double-layer electrode and a pseudocapacitive electrode (Fig. 3a,c)^21^,^29^,^30^,^31^,^32^. In 2001 Amatucci *et al*.^29^ demonstrated that these hybrid ECs can deliver a higher power density than Li^+^ batteries and a higher energy density than a conventional EDLC. Commercially available Li-ion hybrid ECs have gravimetric energy densities of about 20–30 Wh kg^−1^, about 3–5 times higher than that of conventional EDLCs using activated carbon (AC) at both electrodes. The high energy density is due to the added redox capacitance and a wider voltage window^21^. The Li_4_Ti_5_O_12_/AC hybrid EC (Fig. 3a,c), has demonstrated remarkable energy and power densities with extended cycling at extremely high (1,200 C) rates^21^. Another benchmark example is a commercialized hybrid device technology that represents a significant step towards environmentally friendly energy storage devices. The ‘Aquion battery' is, in fact, a spinel-MnO_2_/AC hybrid EC that uses sodium sulfate solution as electrolyte. This technology provides excellent cycling performance (no degradation after 10,000+ cycles) and ∼20 Wh kg^−1^ specific cell capacity due to the use of pseudocapacitive λ-MnO_2_ and operation in the 1.6 V window in the aqueous electrolyte^32^,^33^. Hybrids also have successfully been applied to the next generation of lead-acid batteries. By replacing one of the lead plates with an activated carbon electrode, power density was improved by a factor of two and cycle life was extended by more than a factor of three^34^.

The hybrid devices based on traditional planar architectures are limited in the thickness of the pseudocapacitive electrode because of low electrical and ionic conductivities, which slow the overall device kinetics. Both ionic and electronic conductivities are critical for increasing rate performance of battery electrodes, especially when large and multivalent ions are used in electrolytes^35^. As a result, the energy density of EES materials in thick electrodes is much lower than the theoretical values that can be achieved with very thin films. Thus, an important goal for EES electrodes is co-integration of different materials at the nanoscale and the development of new material architectures that are scalable and enable rapid ion access to each electrochemically active site. As discussed in the following section, new approaches to electrode design need to be implemented to achieve fast energy storage combined with high energy density and a long cycling lifetime for devices. In particular, a transition to 3D nanoarchitectures is required to improve ion access to electrochemically active surfaces and minimize both diffusion limitations as well as macroscopic electrode strain during charging/discharging. 3D architectures also allow the use of thicker electrodes. As a general strategy, the use of nanostructured materials^36^ allows us to increase ion accessibility and rate performance owing to their high surface-to-volume ratio.

## Hierarchical and three-dimensional structures

In the first generation of electrode architectures, researchers emphasized homogeneous nanomaterials, such as nanoparticles (0D), nanowires and nanotubes (1D), layered materials (2D) and mesoporous structures (3D). Nanoparticles of various chemical compositions have demonstrated great potential for high-rate energy storage. For typical Li-ion battery materials, such as LiCoO_2_, Si, Ge and so on, nanoparticles lead to substantial improvement in power performance and cycling behaviour due to shorter ion diffusion paths and accommodating Li insertion with minimal internal strain^37^,^38^,^39^. In the case of EDLCs, superior power density at charging rates as high as 10 V s^−1^ was demonstrated for micro-supercapacitors based on carbon onions that are 6–7 nm in diameter^40^. 1D nanostructures (nanowires, nanotubes) offer most of the benefits of the nanoparticles and, moreover, they can be assembled into free-stranding and flexible electrodes without addition of binder due to their high aspect ratio^39^,^41^,^42^. Conductive carbon nanotubes are used extensively as an additive to conventional battery electrodes to decrease electrode resistance and mitigate associated Ohmic losses, as well as to improve the rate performance of devices^43^.

**Figure 4:**
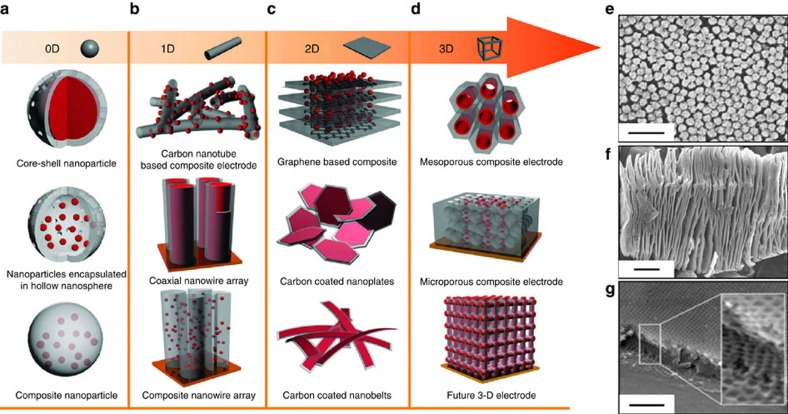
Three-dimensional nanostructures. Schematic of heterogeneous nanostructures based on 0D (**a**), 1D (**b**), 2D (**c**), 3D (**d**) structure motifs. Panels (**a**–**d**) reproduced from ref. 51 (Copyright 2011 Royal Society of Chemistry) and pomegranate composite electrode illustration from ref. 52 (Copyright 2014 Nature Publishing Group). Examples of mesoporous architectures. (**e**) CdSe nanoparticles assembly. Reproduced from ref. 95 (Copyright 2011 Nature Publishing Group). (**f**) Ti_3_C_2_T_x_ nanolaminates (MXene). Reproduced from ref. 96 (Copyright 2014 Elsevier Publishing Company). (**g**) Mesoporous Nb_2_O_5_ film. Reproduced from ref. 48 (Copyright 2010 American Chemical Society).

2D and layered materials that can be intercalated and exfoliated are particularly promising for incorporation into electrodes. 2D materials, such as graphene^44^, some transition metal oxides^12^, MoS_2_ (ref. 16) and Ti_2_C (MXene)^45^, are only a few atoms thick and therefore have a large number of immediately accessible electrochemically active sites. For many layered materials or assembled 2D structures, thermodynamic considerations are such that spontaneous intercalation takes place which leads to preferential occupation of sites. Another benefit with 2D materials is that large ions such as Na^+^ and K^+^ as well as solvated ions can be accommodated on their surface or between the layers. Finally, the 2D morphology is also convenient for flexible energy storage materials^46^. Although only limited research has been carried out to date, indications are that the electrochemical performance of 2D metal oxides is substantially better than the corresponding bulk material^46^. Metallic 2D materials such as MXenes^15^ and 1T transition metal dichalcogenides^16^ (Fig. 4c,f) are particularly attractive because they intrinsically possess good electrical conductivity along with the ability to support redox reactions from their transition metal chemistry.

Nanomaterials with templated 3D mesoporosity represent another important category of EES materials. They have a high surface area due to the combination of mesopores generated by the organic template and micropores, which exist between nanocrystals. This combination of surface area and mesoporosity which enables electrolyte penetration throughout the material, leads to a higher capacity and rate capability than with non-templated nanocrystalline materials^47^. Significant improvement in electrochemical performance was observed for several pseudocapacitive metal oxides including Nb_2_O_5_, CeO_2_ and other systems (Fig. 4g)^48^,^49^.

Although nanostructuring provides undeniable benefits, there are still issues that need to be addressed. For transition metal oxides, which are primarily insulators or wide bandgap semiconductors, there is the need to increase electronic conduction by doping, through partial reduction, and by creating good electrical contact between nanoparticles and conductive additives. The restacking of 2D layers, if not carried out properly, can impede ion access to the redox-active sites. This behaviour becomes of greater concern with increasing electrode thickness. Another potential limitation is that the packing density of nanoparticles is low and conventional rolling or doctor-blade electrode manufacturing techniques produce electrode films with a low density. As a result, electrodes comprised of just nanotubes or nanowires may not achieve satisfactory volumetric characteristics.

To address these limitations, approaches that use composite electrode materials are being pursued. On the one hand, there is the simple mixing of active material with conductive additives such as carbon black, nanotubes or graphene. More sophisticated structures have been developed (Fig. 4a–d), where one material serves as a conductive backbone for the electrochemically active material, thus providing structural integrity and mechanical stability, in addition to enhancing the electronic conductivity of the electrode. Ideally, well-designed composite electrodes should provide synergistic benefits in terms of electrochemical and mechanical properties. The latter are especially important for current collector-free and wearable devices^50^. Poor mechanical strength also leads to faster electrode (and device) degradation^1^.

An interesting example of this multi-functionality is shown with high volume expansion materials such as Si and Ge. The development of 0D core-shell nanoparticles and 3D ‘pomegranate' structures from them, where carbon serves as a protecting and electron-conducting shell, (Fig. 4a,d) were proven to be particularly effective in improving cycle life. In this case, active material nanoparticles are able to ‘breathe' inside the carbon shell during charge–discharge without deterioration of the electrochemical properties^51^,^52^ 1D composite structures that use a similar approach have been designed (Fig. 4b)^51^. Highly conductive 2D materials, such as graphene or MXene, can serve as a backbone decorated by nanoparticles^22^ or alternatively some ‘spacers' can be integrated between the 2D layers to avoid restacking and improve electrochemical performance^51^,^53^. Metal oxide nanoparticles encapsulated in conductive mesoporous carbon templates serve as an example of a 3D composite structure^54^.

In the future, it can be expected that homogeneous nanomaterials will slowly give way to the development of non-planar architectures, at both the materials and system levels, as it has already been demonstrated for carbon^55^,^56^. Nanomaterials will serve as building blocks, which assemble into hierarchical 3D architectures. This structure will improve electron and ion accessibility to redox-active sites, thus enabling fast electrochemical reactions (Fig. 4d, bottom) without introducing excessive porosity that may reduce the volumetric energy density. 3D electrode architectures maximize the number of active sites accessible to ions during charge/discharge and lead to high energy density without compromising power density. Such structures could also incorporate highly conductive components and eliminate the need for current collectors, thereby minimizing volume/mass of inactive materials in the EES device.

## Harnessing multi-electron redox processes

**Figure 5:**
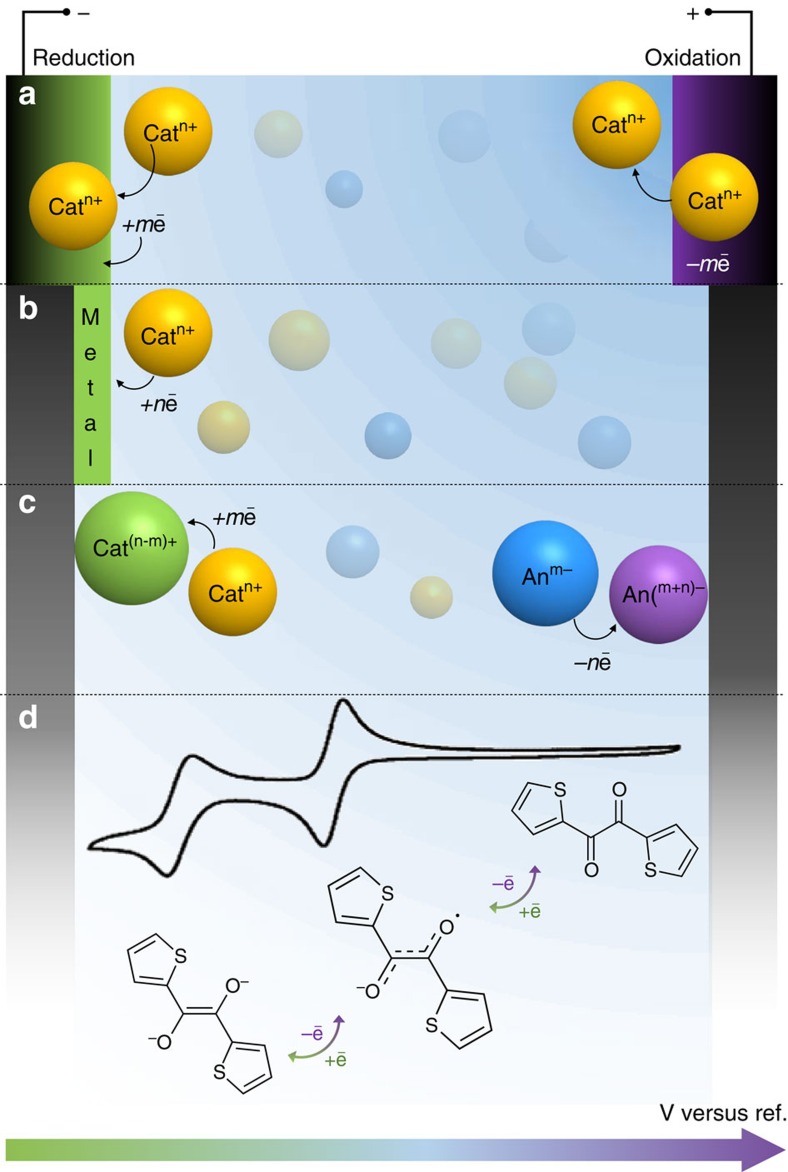
Multi-electron redox processes. (**a**) Insertion materials: change in electrode oxidation state (+/− *m*e^−^) is accompanied by cation (Cat) insertion/extraction. Black colour signifies electrode material in its original state, green colour stands for reduced state (+*m*e^−^), and violet colour stands for oxidized state (−*m*e^−^). (**b**) Rechargeable metal anodes: charge-carrying cations (orange spheres) are reversibly reduced to metal (green block) on the negative electrode, (**c**) charge-carrying ions change their oxidation state without conversion to solid phase. Left side of the schematic stands for the processes related to cations which get reduced by *m*e^−^ on the negative electrode; right side of the schematic illustrates generalized processes that take place on the positive electrode, when anions (An) are oxidized losing *n*e^−^. (**d**) Sample cyclic voltammogram in electrolyte containing organic molecules with multiple redox-active groups.

Progress in electrode architectures provides us with the opportunity to use materials and electrolytes that utilize reversible multi-electron reactions that can markedly increase the energy density by storing/exchanging more than one electron per redox centre^57^. We highlight below the electrochemical cell components that can use multi-electron processes, as well as examples of existing and emerging systems (Fig. 5).

### Cathodes

**Figure 6:**
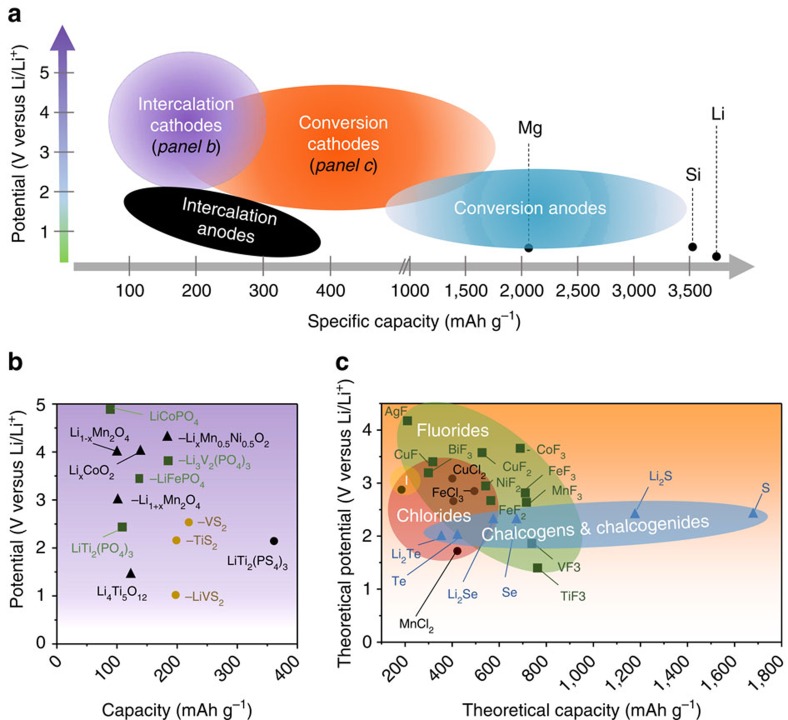
Capacities and operation potentials of different types of electrode materials for Li-ion batteries. (**a**) Overview of the average discharge potentials and specific capacities for different types of electrodes. Adapted from ref. 59 (Copyright 2014 Elsevier Publishing Company). (**b**) Intercalation-type cathodes (experimental). Adapted from ref. 58 (Copyright 2010 American Chemical Society). (**c**) Conversion-type cathodes (theoretical). Adapted from ref. 59 (Copyright 2014 Elsevier Publishing Company).

Research progress in developing new positive electrodes (cathodes) for lithium-ion batteries lags behind the development of negative electrodes, where values in excess of 1,000 mAhg^−1^ are readily achieved (Fig. 6a,b)^58^,^59^. Below, we summarize research directions that aim to push the capacities of cathodes to much higher values.

Intercalation cathodes serve as host networks, where ions can be reversibly inserted and extracted. Typical insertion compounds used are transition metal oxides, metal chalcogenides and polyanion framework compounds, which provide stable performances and are currently used in commercial Li-ion batteries^59^. One of the major limitations is the number of charge-carrying cations that can be inserted into the crystal structure and thus moderate specific capacity (Fig. 6b). Among the systems being investigated are layered Li_2_MeO_2_ (where Me=Co, Ni and Mn) and Li_2_VOPO_4_ (ref. 60), where two Li ions can be accommodated. With Li_2_MeO_2_, the Li ions occupy tetrahedral sites between the MeO_2_ layers. On extraction of Li^+^, there is a two-phase reaction to the LiMeO_2_ phase, with the remaining Li^+^ staying in octahedral sites between the layers. The major drawback is that subtle shifting of the layers impacts the long-term stability of the lattice. As a potential solution, the use of Mg^2+^ instead of Li^+^ can be considered: the layer shifting may be avoided, provided Mg^2+^ intercalates reversibly into only the octahedral sites, as fewer sites are required to transfer the same or larger charge.

Phase changing cathodes are electrode materials that involve breaking and recombination of chemical bonds and generation of multiple phases. These reactions are typically multi-electron and therefore potentially high capacities can be achieved. Currently, cathode systems that are promising in terms of theoretical capacities and operation potentials are: (i) transition metal halides, such as chlorides and fluorides, and (ii) chalcogens and chalcogenides, for example the Li–S system offers a high theoretical capacity of 1,675 mAh g^−1^ combined with low cost (Fig. 6c)^59^,^61^. Utilization of the phase changing multi-electron systems in both positive and negative electrode materials offers the opportunity for a transformative impact on EES by more than doubling the theoretical gravimetric energy density compared with currently used intercalation systems. For instance, the theoretical energy density for the Li–S system is 2,567 Wh kg^−1^ (or 2,199 Wh l^−1^ based on the sum of the volumes of Li at the beginning and Li_2_S at the end of discharge)^61^. It is important to remember that to enable multi-electron systems to operate efficiently it is essential to apply 3D nanostructuring of electrodes as discussed above to achieve reliable and fast performance of the devices with good cyclability. In the case of Li–S batteries (which suffer from high volume changes, low conductivities and dissolution of the reaction intermediates, polysulfides) an efficient strategy to boost performance involves encapsulation of the sulfur nanoparticles in 3D porous and conductive structures, that is, carbon^62^, MXenes^63^,^64^ and so on, to provide adequate electronic transport for the reaction and to minimize the capacity losses arising from self-discharge effects.

### Rechargeable metal anodes

Non-lithium metal anodes (such as Al, Mg and so on) can be of interest due to the possibility of multi-electron transfer during metal plating (Fig. 5c). In particular, non-lithium metal anodes, such as Mg in Mg-ion batteries, offer the promise of dendrite-free metal deposition, which would mitigate some safety and durability concerns associated with cell shorting from dendrites piercing through the separator as well as from irreversible metal consumption^65^.

Thus, in Mg-ion batteries it is potentially possible to take advantage of its gravimetric energy density (2,206 mAh g^−1^) and volumetric energy density of 3,833 mAh cm^−3^, which is higher than that of lithium (2,046 mAh cm^−3^). However, even when no dendrite growth occurs, nanostructuring or scaffolding of the pure metal anodes may be required to achieve high charge/discharge rates and avoid large volume changes, sacrificing some of the volumetric energy density. Currently, a key consideration with Mg anodes is the electrolyte system, and development of efficient and noncorrosive electrolytes has been an active research area. In recent work, very promising halogen-free, noncorrosive chemistry for electrolytes was reported with coulombic efficiencies of 99% for Mg plating and stripping^66^. Another interesting strategy suggested for Mg-ion electrolytes is addition of a lithium salt which increases conductivity of the electrolyte, and at the same time enables use of Li-ion cathode chemistries, and improves cycling markedly^64^,^67^.

### Alloying anodes

Elemental anodes such as Si, Sn, Ge and their alloys can offer high capacities, but as mentioned above they do suffer from high volume expansion during charging. Therefore, just as for phase changing cathodes, the composite 3D architectures such as pomegranate-like encapsulation in carbon shells should be applied for their use in energy storage devices that require good cycling behaviour.

### Transition metal compounds

There has been limited study of negative electrodes for multivalent ions based on transition metal compounds. There are a number of transition metal oxides that offer a range of oxidation states (Nb_2_O_5_, TiO_2_ and so on) but the ability to reversibly insert/remove ions such as Mg^2+^ has only been reported for MnO_2_ (ref. 68) and V_2_O_5_ (ref. 69). Instead, it would seem that carbides and 1T-dichalcogenides^16^ are worth considering because of their multiple oxidation states and significant electrical conductivities.

### Redox-active electrolyte

It should also be appreciated that electrolytes possessing redox activity can provide a significant increase in device energy density. Redox-active electrolytes, which are commonly referred to as anolytes and catholytes depending on whether the redox process in the electrolyte is taking place on the positive or negative electrode, can be divided into two groups: inorganic and organic.

### Inorganic electrolyte

A typical inorganic electrolyte or an additive to electrolytes contain ions that can change their oxidation state within a suitable potential window. The addition of potassium iodide to an aqueous electrolyte increased the capacitance of the high SSA carbon electrode by an order of magnitude: from 100 F g^−1^ to 1,200 F g^−1^. The reason is that an iodide ion reversibly loses up to 6e^−^ following the electrochemical reaction (equation (1)),


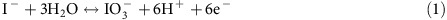


in the potential range of ∼0.12 V. In a similar manner, a vanadyl cation (VO^2+^) can gain 3e^−^ and produce capacitance of 670 F g^−1^ in the potential range of ∼0.7 V. It is important to mention that use of the electrolytes featuring multi-electron redox requires an ion separation membrane to prevent mixing of the electrochemically active species and self-discharge^70^. Also, such substantial changes in the iodine oxidation state are possible in aqueous electrolytes, whereas in organic electrolytes iodide ions can gain only 1e^−^ per iodine atom^71^. Transition metal oxides that can change their oxidation state while staying in solution (for example, Ce^3+^ to Ce^4+^), may also be attractive^72^. Use of redox-active electrolytes can compensate for the energy density lost due to the development of open electrode architectures that promote ion accessibility.

### Redox-active organic molecules

Electro-active organic materials possessing multiple redox groups offer considerable potential for surpassing present secondary battery performance^73^. Organic materials consist of low-cost, lightweight, earth-abundant elements and their properties can be rationally tuned using well-established principles of organic chemistry. Organic redox materials offer the added advantage that structural changes associated with the redox reactions are generally small^74^,^75^. Molecules such as quinones are simple and tuneable through synthesis. In nonaqueous solvents, quinones can be reduced reversibly in two one-electron steps to the anion radical, equation (2), and di-anion, equation (3), respectively. The difference in formal potentials between these two redox processes is typically on the order of 500 mV.


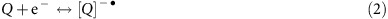



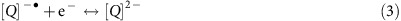


In aqueous media, however, a reversible two-electron, two-proton process (equation (4)) becomes possible at potentials that are shifted positively, typically by hundreds of millivolts. This shift represents an increase in cell voltage for a device incorporating such a redox process.


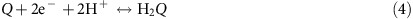


The same effect can be observed in nonaqueous media in the presence of strongly interacting cations, including Li^+^ as well as divalent cations such as Mg^2+^, at potentials that may be as high as 4 V versus Li^75^. Organic Li-oxocarbon salts can also react reversibly with Li. For example, Li_2_C_6_O_6_ reacts with 4 additional Li^+^, albeit with poor cycling behaviour^74^. Similarly, substituted organic molecules containing N, O and S heterocycles can be reversibly oxidized and reduced. These molecules are generally more stable and can show stable and reversible redox activity^76^.

## Recent advances in computations and simulations

**Figure 7:**
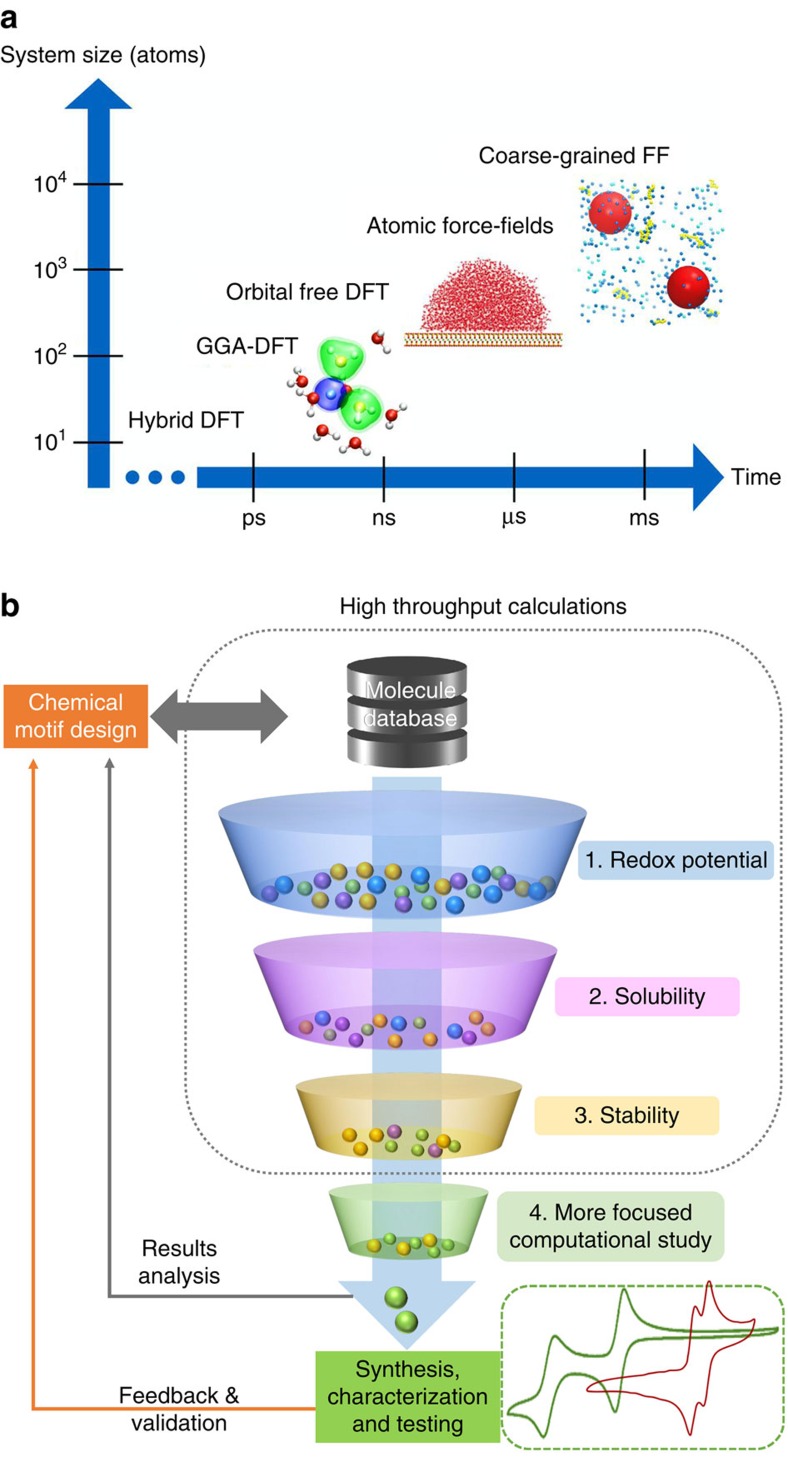
Modelling and simulations. (**a**) Schematic representation of the computational techniques applied depending on system size; increased system complexity results in longer computation times. FF stands for force field. Image is courtesy of M. Salanne, Pierre and Marie Curie University, with snapshots reproduced from ref. 97 (Copyright 2012 American Institute of Physics), ref. 98 (Copyright 2011 American Chemical Society) and adapted from ref. 99 (Copyright 2007 American Institute of Physics). (**b**) Schematic of down-selection of candidate molecules for electrical energy storage applications based on high-throughput computations using quantum chemical calculations of specific properties. Based on screening, selected molecules can be subjected to further focused computational studies and proposed for synthesis and testing. Adapted from ref. 100 (Copyright 2015 American Chemical Society).

It is clear that current energy storage technologies are far from being ideal, and there is a need to redesign the energy storage device in terms of materials, architectures and electrolytes, among other features. To accomplish this goal requires more than just extensive parametric experimental studies. The challenge facing the experimental selection of materials and electrode architectures is that there are too many options available. For example, dozens of new 2D materials of potential use for electrode applications were discovered just in the past few years and many more are predicted to exist^77^,^78^. There are also numerous parameters that can be varied (for example, pore size and shape, number of layers in 2D structures, composition and so on). The parameter space increases markedly when matching materials with appropriate electrolytes is considered. The question of how to select the right electrolyte for a particular material cannot be answered by just trying many thousands of salt solutions in every available solvent^79^. To promote future discoveries and achieve breakthroughs in energy storage there must be close integration of theory, modelling and simulation with synthesis and characterization over the full range of length and time scales*—*from atoms to microstructures to systems (Fig. 7a). Theory and modelling can provide guiding principles for selection of electrode materials, matching electrolytes and design of new architectures.

While density functional theory and molecular dynamics models are primarily used at the nanoscale, finite element modelling can be used to simulate processes that occur at a larger scale than that shown in Fig. 7. Computational studies create new opportunities to understand, design and manufacture electrochemical energy storage systems from the bottom up, based on the fundamental processes governing performance, degradation, cost, efficiency and manufacturability. Computational materials science provides a powerful tool to screen for new materials that might have an optimal combination of transition metal chemistry, conductivity and electrochemical stability. *Ab initio* computations are used for prediction of new chemical compositions of oxides, sulfates and phosphates^80^,^81^ that could yield high capacity, which is particularly important in the search for new cathode materials. Efforts to create the ‘materials genome' have been expanding in the past few years^82^.

First-principles computational methods can be used to study the energy landscape for intercalation and diffusion barriers of materials and thus provide new insights regarding ion transport phenomena, and explain electrochemical characteristics. For example, the origin of the high rate properties of orthorhombic and monoclinic Nb_2_O_5_ was investigated using Monte Carlo simulations and showed that open channels in the network of quasi-2D NbO_x_ effectively reduced the energy barrier for Li^+^ ion diffusion from one site to another^83^. These studies are also likely to provide fundamental insights regarding stress build-up and heat transfer due to phase transformations, and lead to the design of electrodes for high rates and low degradation, and enable high-yield, high-throughput manufacturing. For non-lithium-ion batteries and metal-ion capacitors, where the experimental database is more limited, use of computational techniques allows one to minimize the number of exploratory experiments and select material/ion combinations that offer the most promise.

As mentioned above, 2D materials, such as layered transition metal dichalcogenides, carbides, nitrides and oxides, can naturally accommodate larger ions in between layers and therefore their computational screening is potentially the most rewarding. For example, computations of 2D transition metal carbides (MXenes) using first-principles methods for Na^+^, K^+^, Mg^2+^ and Al^3+^ ions, suggest substantial promise for these materials as anodes for non-lithium-ion batteries^84^,^85^. Another important computational study highlighted the promise of using 2D materials with polar functional groups for Li–S batteries due to their anchoring effect on polysulfides. This behaviour was confirmed in experimental studies^86^.

Simulations of electrode architectures usually require studies at larger length scales and are just beginning to emerge. Meunier *et al*. used computations to investigate 2D and 3D networks of carbon chains connected covalently and evaluated their mechanical properties^87^. This study can guide the engineering of 3D architectures that have a conductive backbone. These and other 3D structures from carbon can be created using a variety of synthetic techniques^88^. Vatamanu *et al*. used molecular dynamics simulations to show that nanoporous structures made of arrays of conductive carbon chains represent a synergy of high curvature, atomic scale roughness and nano-confinement which can generate capacitance levels that greatly exceed the performance of the current generation of materials^89^. These architectures are still to be created.

Computational approaches can also be used to effectively screen new electrolytes (Fig. 7b). The electrolyte genome project is currently investigating the stability and compatibility of different redox molecules for multi-electron electrochemical processes^79^.

## Integrating faradaic and capacitive storage mechanisms

Non-planar electrode architectures may play an important role in future EES systems. Designed to be heterogeneous at the nanoscale, this approach enables devices to benefit fully from multi-electron faradaic processes with minimum diffusion limitations and without phase transformations that hinder kinetics. The prospect of designing architectures with minimal expansion offers a route towards significantly decreasing the volume/mass required for non-active components, since packaging can be simplified and bulky current collectors eliminated. In developing these architectures, not only conventional materials such as carbon and metal oxides and their combination should be considered, but also new materials in reduced dimensionalities that intrinsically possess metallic conductivity and redox-capable chemistries, such as MXenes and 1-T dichalcogenides, should be explored. Concurrently, there is a need to develop new electrolytes that are compatible (no parasitic reactions or corrosion) with all components of the device and can withstand many thousands of cycles with stable performance over a wide voltage window.

**Figure 8:**
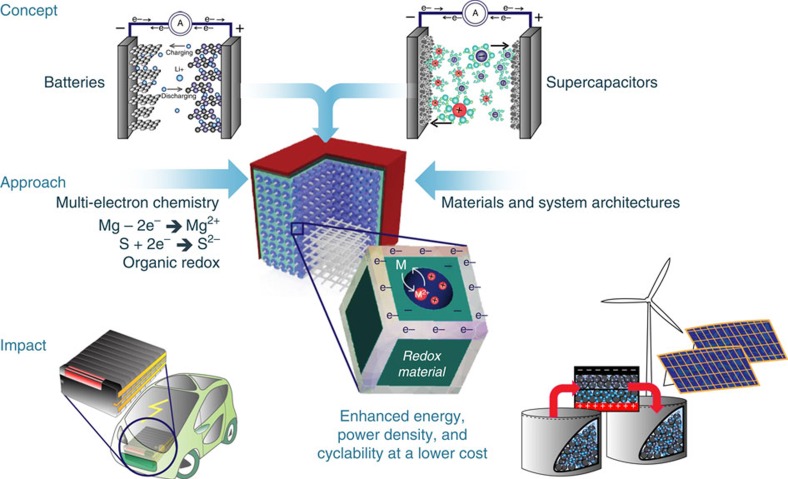
Redefining electrical energy storage. Conceptual presentation of development of fully integrated rechargeable hybrid battery-supercapacitor (supercapbattery) electrical energy storage devices. Image courtesy of K. Jost, Drexel University.

A concept for future energy storage is depicted in the schematic shown in Fig. 8. In the opinion of the authors, future energy storage systems will be hybrid devices combining the best features of metal-ion batteries and ECs. Such devices are based on hierarchical electrode architectures consisting of interconnected thin scaffolds of an active solid-electrode material containing designed micro/mesopores that provide a large surface area in contact with a liquid or gel electrolyte, enabling fast charge–discharge with minimal strain generation. Electrons should ideally be provided from a conductive scaffold forming the electrode backbone, thus all but eliminating current collectors. Utilization of multi-electron chemistries of both electrolyte and electrode materials will increase the amount of energy stored. The impact of these developments may start with portable electronics but will go well beyond, impacting large-scale EES by facilitating storage of electrical energy from renewable sources such as solar and wind.

## Gaps in the current knowledge

As a concluding remark, we separately highlight some of the research directions that in our opinion are essential for the further advancement of EES but are still underdeveloped.

1. Understanding the influence of surface chemistry on the electrochemical properties of materials. On nanostructuring for improved material and device performance, the influence of the surface increases markedly. Can we control capacity levels through the design of surface chemistry?
2. New electrolytes. Relatively few electrolytes are used in conventional Li-ion batteries and supercapacitors, yet once we have created open and accessible electrode structures, electrolytes that contain bulkier ions, such as ionic liquids, can be used effectively. At the same time, the introduction of new energy-storing materials and their designed surface chemistries may require the development of new, compatible electrolytes that operate over a wide voltage window. Efforts should also be directed towards developing new multi-electron redox electrolytes.
3. Self-discharge limits long-term energy storage in capacitive electrodes and leads to irreversible energy losses^90^. However, there is relatively little fundamental understanding of the self-discharge mechanisms and how it can be minimized in pseudocapacitive and hybrid systems, in devices containing redox electrolytes and in open 3D and 2D architectures. In particular, the use of redox electrolytes and open architectures may lead to faster self-discharge and novel approaches to mitigating this effect are necessary. Effects of electrode thickness, dimensionality and chemistry among other factors are likely to be significant and need further study.
4. Mechanisms of performance degradation are poorly understood, especially for new material systems. Understanding how material properties degrade will help to expand the lifetime of EES devices.

The next generation of EES devices should be able to couple the redox reactions that lead to high energy density systems with the high charge/discharge rates exhibited by all-carbon supercapacitors. It has been our intent in this review to describe some pathways which put this exciting goal within our reach. The realization of this new generation will have an enormous impact on our future.

## Additional information

**How to cite this article:** Lukatskaya, M. R. *et al*. Multidimensional materials and device architectures for future hybrid energy storage. *Nat. Commun.* 7:12647 doi: 10.1038/ncomms12647 (2016).
